# Supplementary figures and images for: Nicotine induces abnormal motor coupling through sensitization of a mechanosensory circuit in Caenorhabditis elegans
Source: PLoS Biol. 2025 Oct 3;23(10):e3003423. doi: 10.1371/journal.pbio.3003423 (PMC12507281; doi:10.1371/journal.pbio.3003423)

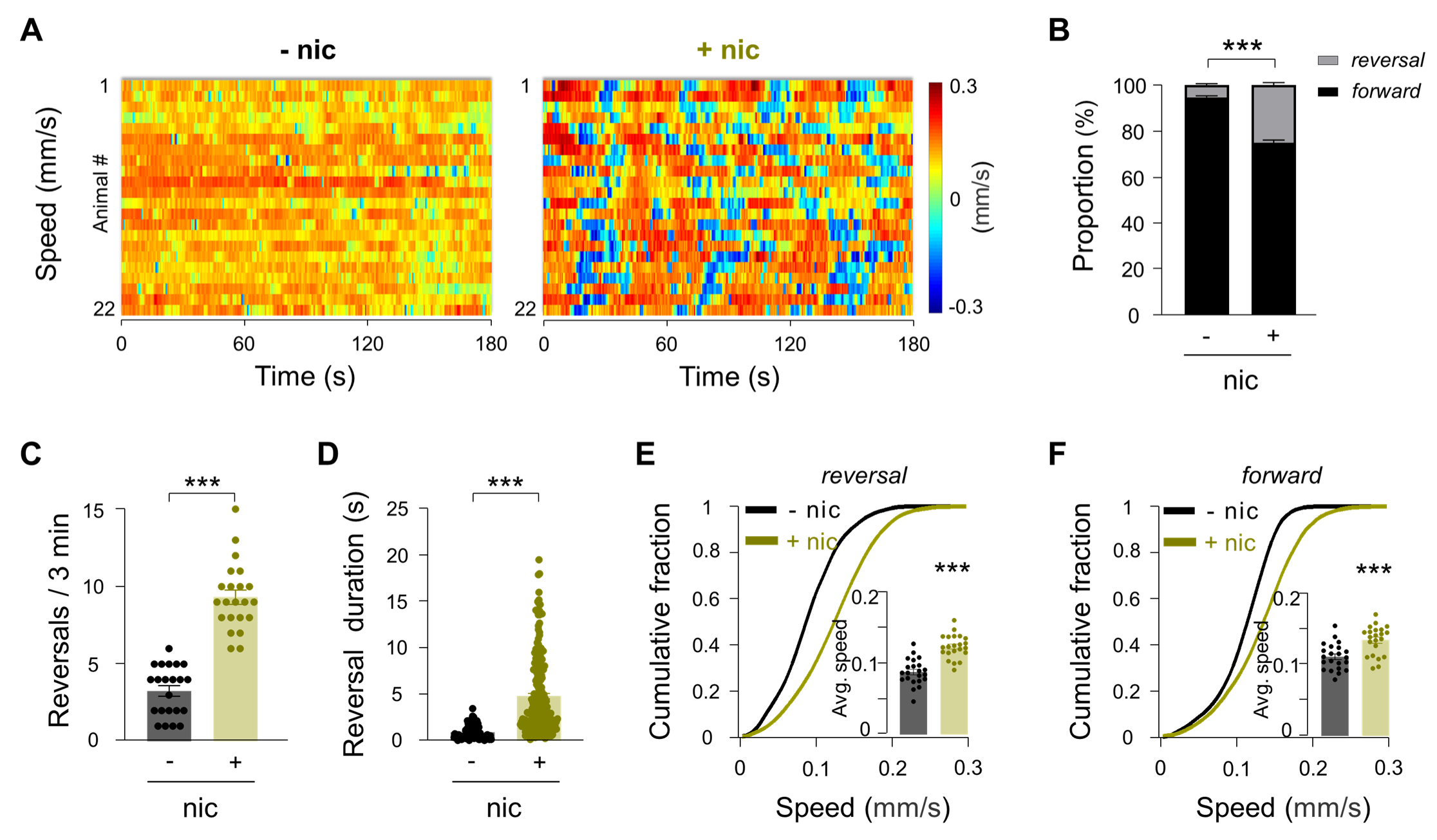

Supplement: S1 Fig — (A) Raster plots displaying individual locomotion speed for animals without (− nic) and with (+ nic) nicotine exposure (n = 22 animals per group). (B) Quantification of the proportion change of forward and reversal movements with nicotine. Nicotine significantly increased the proportion of reversal. *** p < 0.001 by Two-sample Z test. (C, D) Quantification of the reversal frequency and duration changes after nicotine exposure. (E, F) Distribution of instantaneous speed of reversal (E) and forward (F) locomotion. Nicotine leads to a drastic increase of speed in both reversal and forward locomotion. *** p < 0.001 by Student t test. Error bars, SEM. The data underlying this figure can be found in S1 Data. (TIF) [file pbio.3003423.s001.tif]

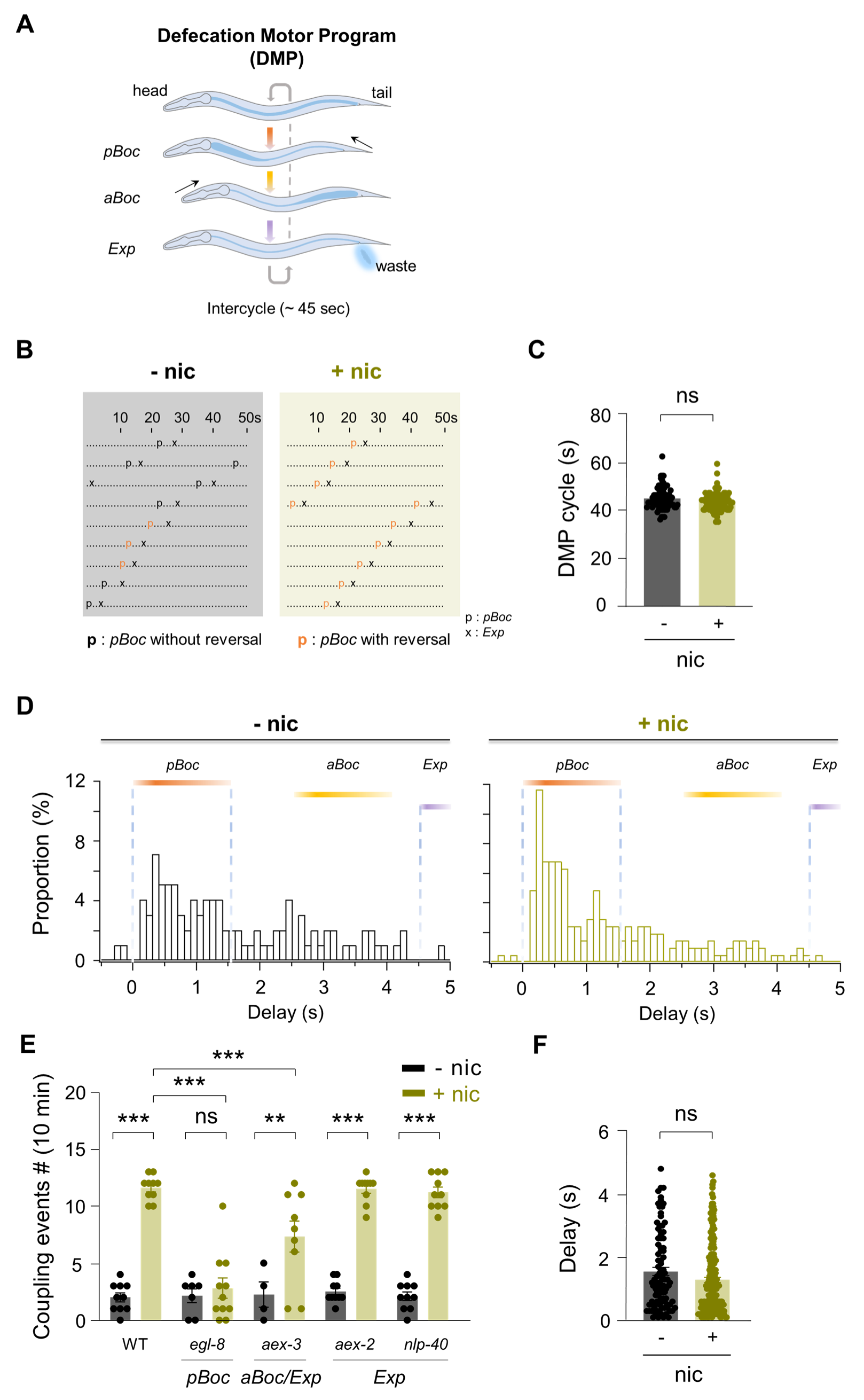

Supplement: S2 Fig — (A) A schematic diagram of C. elegans defecation motor program (DMP). DMP is initiated by posterior body contraction (pBoc), and followed by anterior body contraction (aBoc) after ~2 s relaxation phase and then enteric muscle contraction, leading to expulsion of the gut contents (Exp), and an intercycle of approximately 45 s. (B) Representative ethograms of consecutive 10 defecation cycles in wild-type worms before (− nic) and after (+ nic) the exposure of nicotine (1 mM). Each dot represents 1 s. ‘‘p’’ stands for pBoc and ‘‘x’’ indicates Exp. aBoc is omitted due to difficulties in observation. (C) Quantification of the DMP cycle with or without nicotine exposure. (D) The distribution of the delay time between reversal initiation and different DMP phases. The aBoc is based on estimation. (E) Quantification of the average coupling events in wild-type and respective mutants in 10 min. Two-way ANOVA was performed (interaction: F(4, 81) = 15.82, P < 0.0001). (F) Quantification of the average delay time, which was not affected by nicotine exposure. n ≥ 8 animals (Each dot represents a single DMP cycle). ns, no significance, * p < 0.05, ** p < 0.01, *** p < 0.001 by Two-way ANOVA analysis. Error bars, SEM. The data underlying this figure can be found in S1 Data. (TIF) [file pbio.3003423.s002.tif]

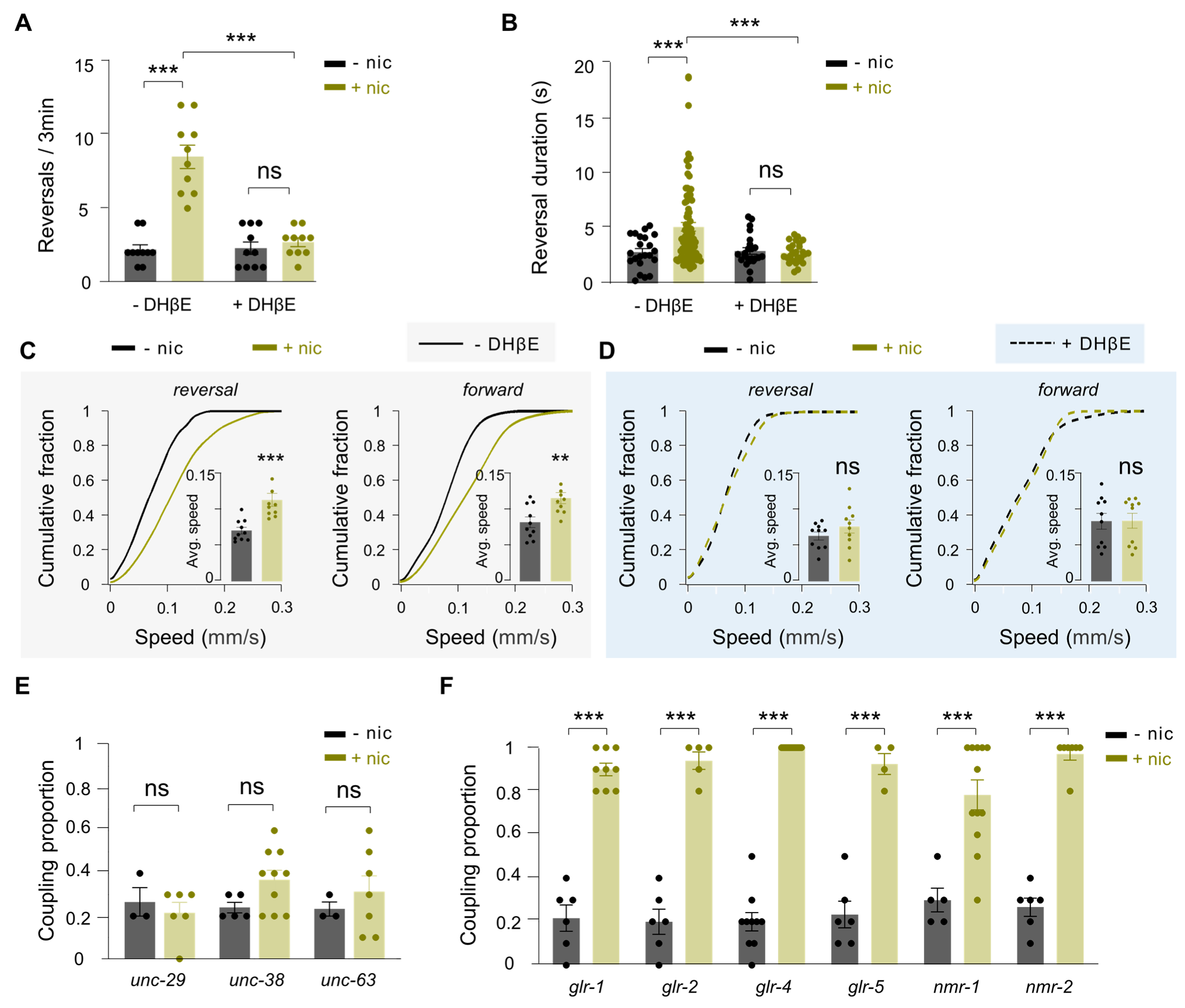

Supplement: S3 Fig — (A) Suppression of nicotine increased reversal frequency by DHβE (20 µM). Two-way ANOVA was performed (interaction: F(1, 36) = 34.69, P < 0.0001). (B) Suppression of nicotine increased reversal duration by DHβE (20 µM). Two-way ANOVA was performed (interaction: F(1, 154) = 5.442, P = 0.0210). (C, D) Distribution of instantaneous speed of reversal and forward locomotion. Nicotine leads to a drastic increase of speed in both reversal and forward locomotion (C). Velocity changes due to nicotine are all inhibited by DHβE (D). ns, no significance, ** p < 0.01, *** p < 0.001 by Student t test. Error bars, SEM. (E) Mutations causing uncoordinated locomotion in nAChR mutants, including unc-29, unc-38, unc-63, suppressed the nicotine exposure induced DMP-reversal coupling. Two-way ANOVA was performed (interaction: F(2, 29) = 1.219, P = 0.3101). (F) No significant suppression of nicotine enhanced DMP-reversal coupling was observed in glutamate receptors mutant worms. Two-way ANOVA was performed (interaction: F(5, 79) = 2.669, P = 0.0279). n ≥ 5 animals. ns, no significance, *** p < 0.001 by Two-way ANOVA. Error bars, SEM. The data underlying this figure can be found in S1 Data. (TIF) [file pbio.3003423.s003.tif]

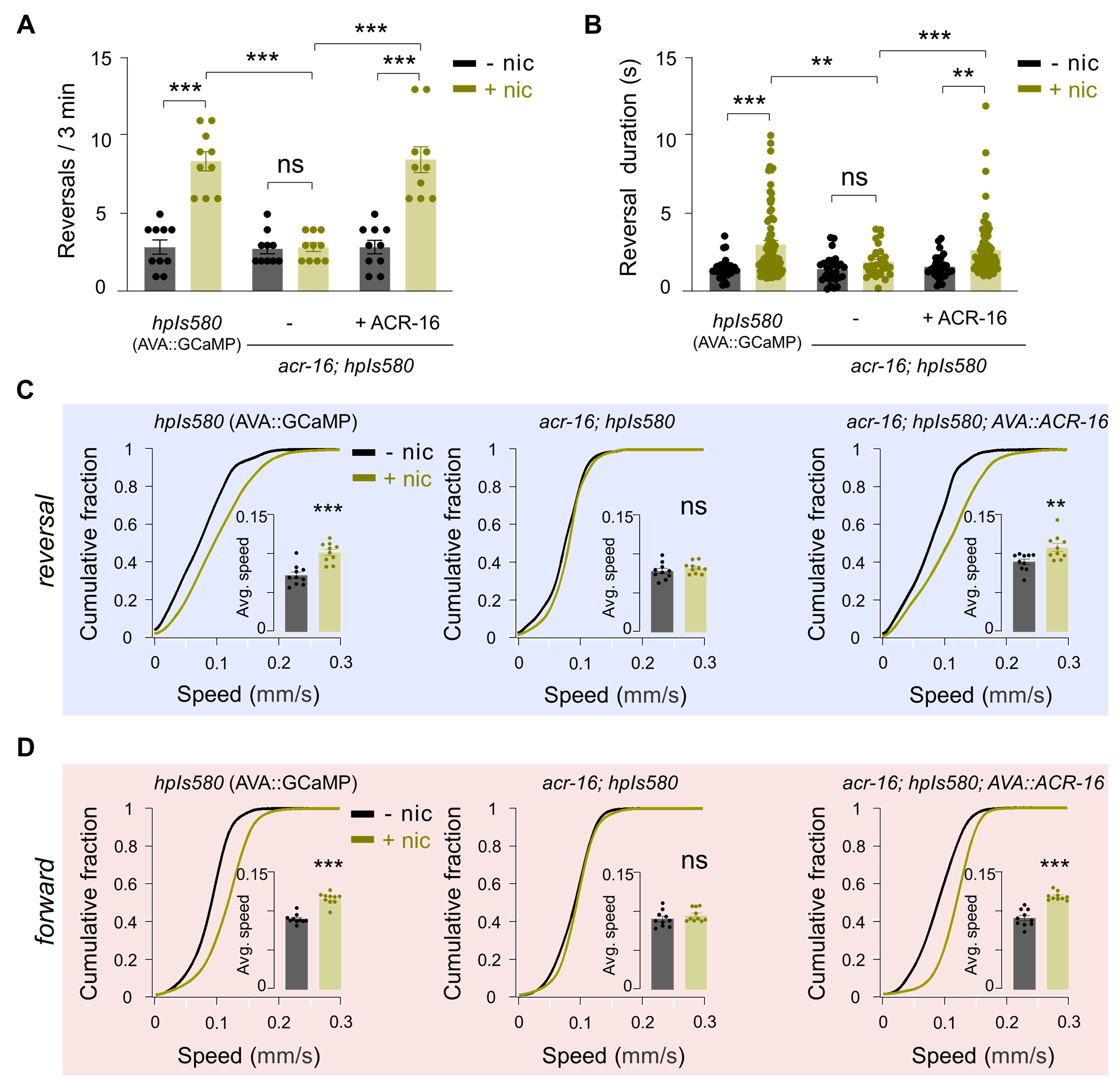

Supplement: S4 Fig — (A) Quantification shows the nicotine enhanced reversal frequency observed in hpIs580 (AVA::GCaMP) worms is abolished in acr-16 mutants. Two-way ANOVA was performed (interaction: F(2, 54) = 17.92, P < 0.0001). (B) Quantification shows that the nicotine enhanced reversal duration observed in hpIs580 worms is abolished in acr-16 mutants. Reversal frequency and duration were restored by expression ACR-16 back. Two-way ANOVA was performed (interaction: F(2, 274) = 1.870, P = 0.1561). ns, no significance, ** p < 0.01, *** p < 0.001 by Two-way ANOVA analysis. Error bars, SEM. (C, D) Distribution of instantaneous speed of reversal (C) and forward (D) locomotion. Nicotine causes a sharp increase in the rate of reverse and forward motion in hpIs580 worms, eliminated by acr-16 mutants. n = 10 animals. ns, no significance, ** p < 0.01, *** p < 0.001 by Student t test. Error bars, SEM. The data underlying this figure can be found in S1 Data. (TIF) [file pbio.3003423.s004.tif]

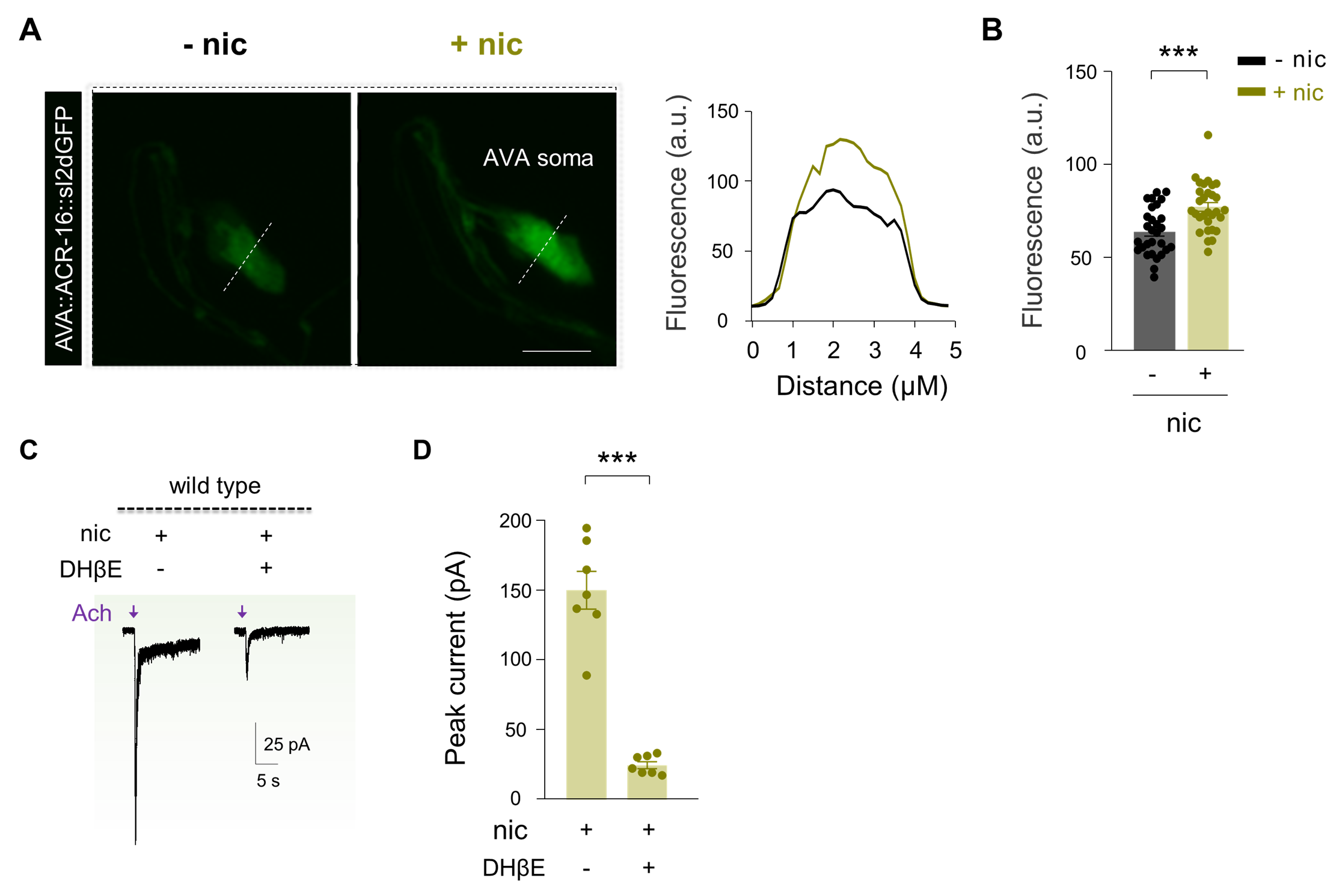

Supplement: S5 Fig — (A) Nicotine up-regulates ACR-16 expression in AVA. Using ACR-16 cDNA with a fluorescent marker (sl2d::GFP) specifically in AVA neurons. (B) After nicotine exposure, the fluorescence intensity in AVA soma is significantly increased. (C) Nicotine-induced increase in I ACh could be suppressed by DHβE (20 µM). (D) Quantification of the peak current with DHβE. n ≥ 6 animals. *** p < 0.001 by Student t test. Error bars, SEM. The data underlying this figure can be found in S1 Data. (TIF) [file pbio.3003423.s005.tif]

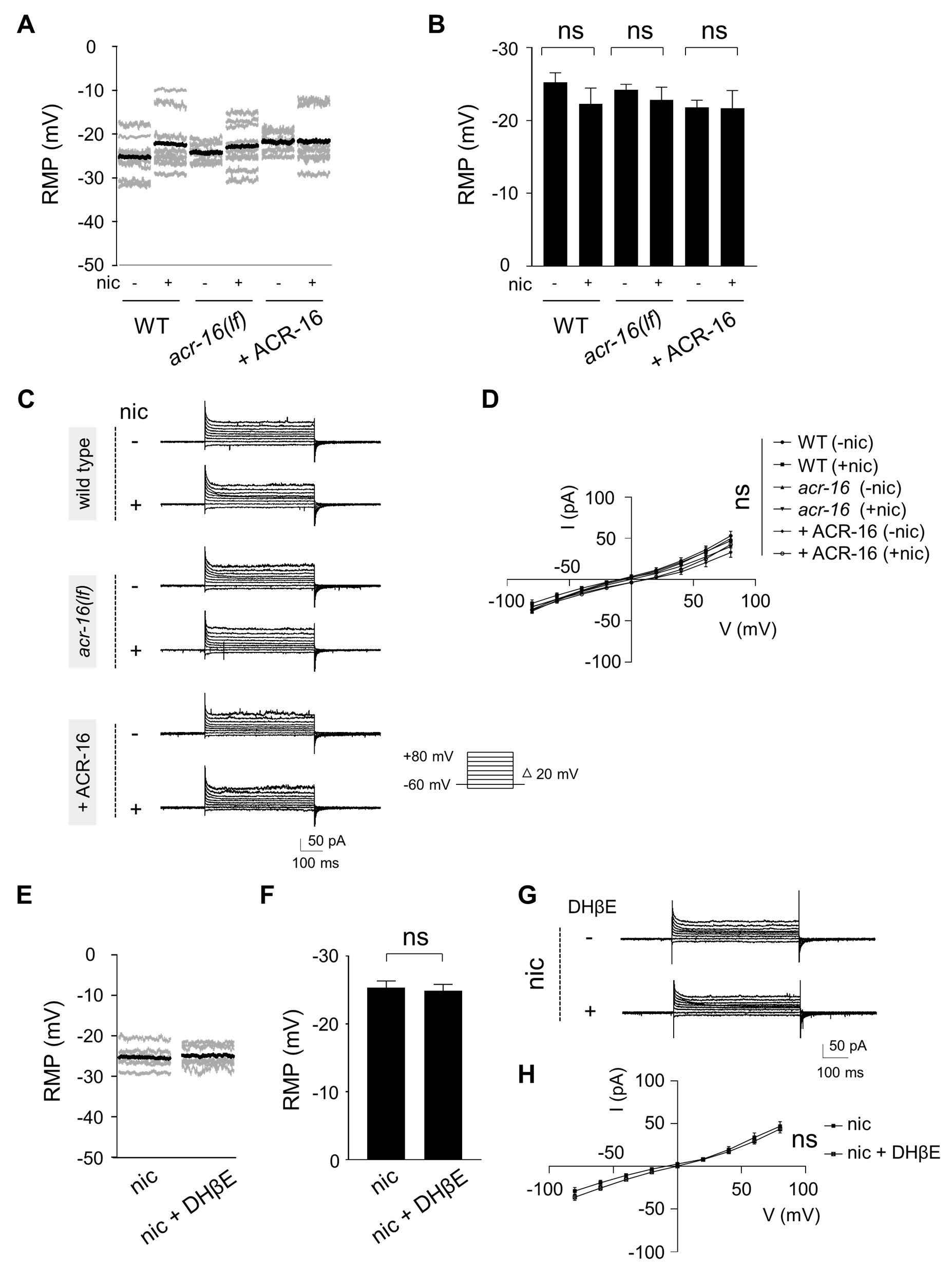

Supplement: S6 Fig — (A) Resting membrane potentials (RMP) of all individual AVA neurons (gray lines) and the average RMP (black lines). (B) Quantification shows that the RMP of AVA exhibit no significantly change in different genotypes before and after nicotine exposure (1 mM, 3 h). Two-way ANOVA was performed (interaction: F(2, 42) = 0.3219, P = 0.7265). ns, no significance, by Two-way ANOVA analysis. Error bars, SEM. (C) Representative step currents in AVA neurons from different genotypes. The currents were evoked by voltage clamp from −80 mV to + 80 mV at 20 mV increment. (D) Quantification of the I-V curve of the voltage-dependent currents. n ≥ 6 animals. (E–H) DHβE (20 µM) had no effect on either the RMP (E, F) or the step currents (G, H) in AVA neurons. ns, no significance, by Student t test. Error bars, SEM. The data underlying this figure can be found in S1 Data. (TIF) [file pbio.3003423.s006.tif]

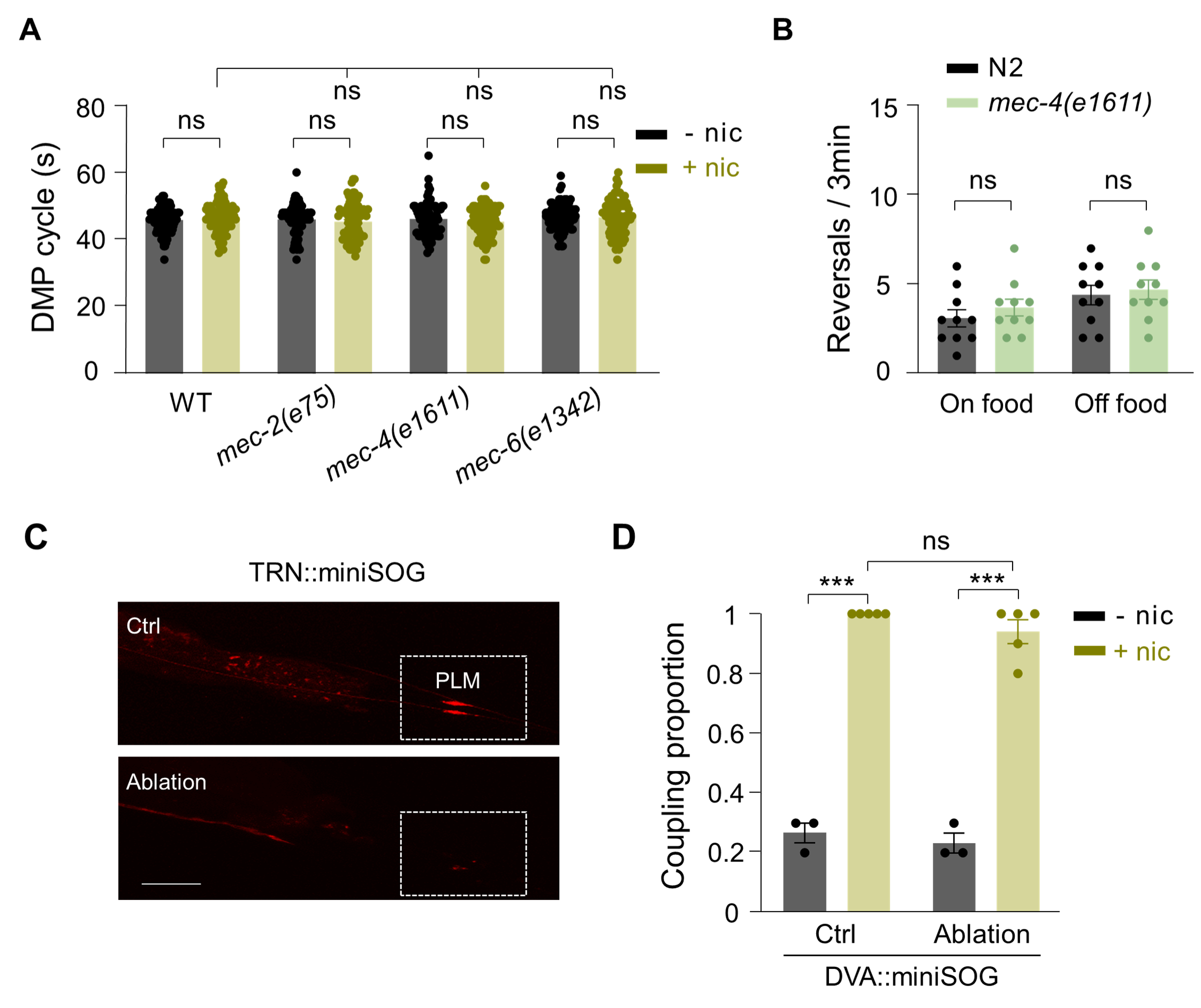

Supplement: S7 Fig — (A) DMP cycle in wild-type N2 and mec-2(e75), mec-4(e1611), and mec-6(e1342) mutants. Two-way ANOVA was performed (interaction: F(3, 712) = 1.238, P = 0.2948). (B) Quantification of the spontaneous reversal rate (# number of reversals per 3 min) in the presence (On food) and absence (Off food) of food, for wild-type (N2) and mec-4(e1611) mutants. Two-way ANOVA was performed (interaction: F(1, 36) = 0.08663, P = 0.7702). (C) Representative TRNs (PLM) before and after the ablation. (D) Ablation of DVA did not change the nicotine enhanced DMP-reversal coupling proportion. Two-way ANOVA was performed (interaction: F(1, 12) = 0.1765, P = 0.6818). ns, no significance, *** p < 0.001 by Two-way ANOVA analysis. Error bars, SEM. The data underlying this figure can be found in S1 Data. (TIF) [file pbio.3003423.s007.tif]
