# Supplementary material for: Nicotine induces abnormal motor coupling through sensitization of a mechanosensory circuit in Caenorhabditis elegans
Source: PLoS Biol. 2025 Oct 3;23(10):e3003423. doi: 10.1371/journal.pbio.3003423 (PMC12507281; doi:10.1371/journal.pbio.3003423)
Supplement: S1 Table — Genotypes used in each figure. (PDF) [file pbio.3003423.s008.pdf]

Table S1. *C. elegans* strains used in this study

Strain	Genotype	Source
WT	N2 Bristol	CGC
FX03342	*acr-2(tm3342)X*	NBRP
RB1659	*acr-3(ok2049) X*	CGC
NC293	*acr-5(ok180) III*	CGC
RB2294	*acr-6(ok3117) I*	CGC
FX863	*acr-7(tm863) II*	CGC
RB1195	*acr-8(ok1240)X*	CGC
VC649	*acr-9(ok933)X*	CGC
FX02515	*acr-10(tm2515) X*	NBRP
RB1263	*acr-11(ok1345)I*	CGC
VC188	*acr-12(ok367) X*	CGC
RB1132	*acr-14(ok1150) II*	CGC
RB1172	*acr-15(ok1214) V*	CGC
RB918	*acr-16(ok789) V*	CGC
RB1226	*acr-18(ok1285)V*	CGC
VC491	*acr-19(ok967) I*	CGC
VC1598	*acr-20(ok1849)/mT1II;+mT1[day-10(e128)]III*	CGC
RB1250	*acr-21(ok1314) III*	CGC
FX04472	*acr-23(tm4472)V*	NBRP
FX03863	*lev-1(tm3863)IV*	NBRP
ZZ15	*lev-8(x15) X*	CGC
TU1747	*deg-3(u662) V*	CGC
TQ24	*xuEx[Pacr-16::ma7 + Pacr-16::yfp2 + Podr-1::rfp]; acr-16 (ok789)*	X. Z. Shawn Xu lab
CB1072	*unc-29(e1072) I*	CGC
ZZ20	*unc-38(x20) I*	CGC
ZZ37	*unc-63(x37) I*	CGC
KP4	*glr-1(n2461) III*	CGC
RB1808	*glr-2(ok2342) III*	CGC
TM3172	*glr-4(tm3172) II*	NBRP
TM3506	*glr-5(tm3506) V*	NBRP
VM487	*nmr-1(ak4) II*	CGC
TM3785	*nmr-2(tm3785) V*	NBRP
ZM9059	*hpIs580 [Prig-3::GCaMP6:wCherry]*	Mei Zhen lab
EN9002	*krSi81[Pmyo-3::TIR1::bfp] I; bab535[acr-16::aid::scarlet::spgfp11x3] V; krEx1404[Pflp-18::lox::STOP::lox::spgfp1-10; Pgap-14::cre; Pmyo-2::mCherry]*	Berangere Pinan-Lucarre Lab
CB75	*mec-2(e75) X*	CGC
CB1611	*mec-4(e1611) X*	CGC
RB1115	*mec-10(ok1104) X*	CGC
FX18782	*trp-1(tm7970) III*	NBRP
TQ194	*trp-2(sy691) III*	CGC
TQ296	*trp-4(sy695) I*	CGC
TU1366	*deg-1(u506) X*	CGC
ST1540	*tmc-1(ok1859B9)*	Lijun Kang Lab
TQ233	*trpa-1(ok999)IV*	CGC
CX10	*osm-9(ky10) IV*	CGC
CB1472	*mec-6(e1342) I*	CGC
QW373	*lite-1(ce314); zfls18[Pmec-4::ChR2::YFP;lin-15+]*	QW Lab
SGA1013	*N2; gaaEx1325[Pacr-16::GFP;Ptwk-40s::RFP]*	This paper
SGA1228	*acr-16(ok789); gaaEx1441[Pnmr-1::ACR-16::wcherry; Plin-44::gfp]*	This paper
SGA1230	*acr-16(ok789); gaaEx1443[Pmyo-3::ACR-16::wcherry; Plin-44::gfp]*	This paper
SGA1232	*acr-16(ok789); gaaEx1445[Punc-4::ACR-16::wcherry; Plin-44::gfp]*	This paper
SGA1229	*acr-16(ok789); gaaEx1442[Psra-11::ACR-16::wcherry; Plin-44::gfp]*	This paper
SGA741	*gaaIs55 N2; [Ptwk-40s::ACR-16::gfp]*	This paper
SGA597	*acr-16(ok789); hpIs580*	This paper
SGA1251	*acr-16(ok789); hpIs580;gaaEx1449[Ptwk-40s::ACR-16::wcherry; Plin-44::GFP]*	This paper
SGA728	*N2; gaaEx1186 [Ptwk-40s::gfp::3'UTR]*	This paper
SGA791	*acr-16(ok789); gaaEx1212 [Ptwk-40s::gfp::3'UTR]*	This paper
SGA1252	*acr-16(ok789); gaaEx1450 [Ptwk-40s::gfp::3'UTR; Ptwk-40s::ACR-16::wcherry]*	This paper
SGA1253	*zfls18; gaaEx1451 [Ptwk-40s::gfp::3'UTR]*	This paper
SGA1254	*acr-16(ok789);zfls18; gaaEx1452 [Ptwk-40s::gfp::3'UTR]*	This paper
SGA1255	*acr-16(ok789); zfls18; gaaEx1453 [Ptwk-40s::gfp::3'UTR; Ptwk-40s::ACR-16::wcherry]*	This paper
SGA937	*N2; gaaEx1322[Pmec-4::miniSOG::wcherry]*	This paper
SGA955	*N2; gaaEx1323[Pmec-4::gtACR2::GFP]*	This paper
SGA1256	*mec-6(e1342); gaaEx1454[Pmec-4::mec-6::GFP]*	This paper
SGA814	*N2; gaaEx1222 [Pnlp-12::miniSOG::GFP]*	This paper
MT1083	*egl-8(n488) V*	CGC
JT5	*aex-3(sa5) X*	CGC
JT3	*aex-2(sa3) X*	CGC
NM4394	*nlp-40(tm4085) I*	NBRP
