# Supplementary material for: Nicotine induces abnormal motor coupling through sensitization of a mechanosensory circuit in Caenorhabditis elegans
Source: PLoS Biol. 2025 Oct 3;23(10):e3003423. doi: 10.1371/journal.pbio.3003423 (PMC12507281; doi:10.1371/journal.pbio.3003423)
Supplement: S2 Table — List of all plasmids used to generate transgenic lines. (PDF) [file pbio.3003423.s009.pdf]

**Table S2. The plasmids used in this study**

| Plasmid | Alias | source |
|---|---|---|
| PSG2 | *pDONR221* | This paper |
| PSG4 | *PDESTR4-R3II* | This paper |
| PSG5 | *Plin-44 :: GFP* | This paper |
| PSG713 | *Pmyo-3 :: acr-16 :: wcherry* | This paper |
| PSG714 | *Punc-4 :: acr -16 :: wcherry* | This paper |
| PSG715 | *Pnmr-1 :: acr -16 :: wcherry* | This paper |
| PSG716 | *Psra-11 :: acr -16 :: wcherry* | This paper |
| PSG717 | *Ptwk-40s :: acr -16 :: sl2dGFP* | This paper |
| PSG718 | *Ptwk-40s :: acr -16 :: wcherry* | This paper |
| PSG719 | *Ptwk-40s ::GFP ::unc-54-3'UTR* | This paper |
| PSG721 | *Pacr-16 :: GFP :: unc-54-3’UTR* | This paper |
| PSG722 | *Ptwk-40s :: RFP :: unc-54-3'UTR* | This paper |
| PSG723 | *Pmec-4 :: miniSOG :: wcherry* | This paper |
| PSG724 | *Pmec-4 :: GtACR2 :: sl2dGFP* | This paper |
| PSG725 | *Pmec-4 :: mec-6 :: sl2dGFP* | This paper |
| PSG726 | *Pnlp-12 :: miniSOG :: sl2dGFP* | This paper |
