# Supplementary material for: Nicotine induces abnormal motor coupling through sensitization of a mechanosensory circuit in Caenorhabditis elegans
Source: PLoS Biol. 2025 Oct 3;23(10):e3003423. doi: 10.1371/journal.pbio.3003423 (PMC12507281; doi:10.1371/journal.pbio.3003423)
Supplement: S3 Table — Primers utilized for PCR amplification to identify mutants and construct transgenic strains. (PDF) [file pbio.3003423.s010.pdf]

Table S3. The primers used in this study

DNA fragment	PCR product length	**Sequence of primers** (F, forward; R, reverse)
Pacr-16	3.5 kb	F: CATTCACAACATATACGGTG
		R: AGTGAATGTACATCCG
Ptwk-40s	1.5 kb	F: TCTAATCACTATCACGTGGG
		R: TGAATATTCATCACTCGATA
Pnmr-1	5 kb	F: CATCTGTTCAGAATTGAGATGC
		R: CGAGGTAGTAGAAGGATGGA
Psra-11	2 kb	F: ACAATGGCTTCATTCAGCG
		R: CGAGGTAGTAGAAGGATGGAA
Pmyo-3	2.3 kb	F: TACTCTATCACTGCCGGC
		R: GATGGATCTAGTGGTCGTGG
Punc-4	3 kb	F: CTGAAACAATTGGTCTACAATCC
		R: GATGTGATTTCAACGTCCC
Pmec-4	1 kb	F: GTACAAGCTTCAATACAAGCTC
		R: GACATTCTATAACTTGATAGCGAT
Pnlp-12	400 bp	F: ATTCTCTTCGTCTTCATCGC
		R: GGAGCATTTTGTCGGAGGC
acr-16 (cDNA)	1497 bp	F: ATGTCTGTCTGCACCCTTC
		R: TTAGGCGACAAGATACGGTG
mec-6 (cDNA)	1134 bp	F: ATGGGTCTCCAATCGGC
		R: CTATGTAATATATGAATGCGTAAGATC
acr-16	1857 bp	F: GTATTCCAGACATGGAACGAC
		R: CGCAAAATGTTACGTCTGG
