# Supplementary material for: Nicotine induces abnormal motor coupling through sensitization of a mechanosensory circuit in Caenorhabditis elegans
Source: PLoS Biol. 2025 Oct 3;23(10):e3003423. doi: 10.1371/journal.pbio.3003423 (PMC12507281; doi:10.1371/journal.pbio.3003423)
Supplement: Attachment — Submitted filename: Reponse letter_R1 (PBIOLOGY-D-25-01396).docx [file pbio.3003423.s016.docx]

REVIEWS:

Reviewer #1: This study by Liu et al. investigates how nicotine exposure alters behavior in worms. The authors discover that nicotine significantly increases reversal frequency and duration during locomotion, and these reversals become tightly coupled with the defecation motor program (DMP). This behavioral change depends on heightened activity in the AVA command interneurons, mediated by upregulation of the nicotinic acetylcholine receptor ACR-16. Furthermore, the effect requires mechanosensory input from touch receptor neurons (TRNs). Functional assays demonstrate that nicotine-exposed animals exhibit enhanced synaptic strength in the TRN-to-AVA circuit, suggesting a novel form of sensitization. Mechanistically, the authors conclude that nicotine upregulates ACR-16 expression in AVA, increasing acetylcholine-evoked currents and AVA calcium activity. These changes sensitize AVA to mechanical input from TRNs. Genetic and optogenetic experiments confirm that both ACR-16 and functional TRNs are essential for nicotine-induced motor coupling. Overall, this is an intriguing study that reveals a surprising role for a classical touch circuit in mediating internal state changes following nicotine exposure. The methodology is rigorous. The conceptual link between nicotine, sensory-motor coupling, and interoception is thought-provoking.

**Response:**

Thank you for your comprehensive and insightful summary, as well as for your positive recognition of the innovative conclusions.

In my opinion, however, there are a few gaps in the study that need to be addressed:

1) The central model proposed in this manuscript is that nicotine enhances AVA activity and reversal behavior through TRN-mediated mechanosensory input. However, the mechanism by which TRNs influence AVA remains unclear. As the authors themselves note, TRNs are glutamatergic neurons, while AVA responds to nicotine in an ACR-16-dependent, cholinergic manner. I appreciate that the authors suggest a cholinergic relay neuron may link TRNs to AVA, but no such neuron is identified or tested. Without direct evidence for this relay, the conclusion remains incomplete. This gap could significantly impact the interpretation of the main findings and should be addressed more directly.

**Response:**

Thank you for your insightful comment. We agree that elucidating the mechanism by which TRNs influence AVA is a key question in understanding the circuit logic. Although the definitive identification of the relay neuron may beyond the scope of the current study—given the lack of specific markers and tools to precisely manipulate all possible intermediaries—we believe this does not diminish the central conclusion of our work: nicotine sensitizes TRN-mediated input to enhance AVA activation and drive reversal behavior. This model remains well-supported by the calcium imaging, pharmacological, and electrophysiological data.

We appreciate this critical observation and have now expanded the ‘Discussion’ to explore this point more directly and in greater depth: “Since TRNs are glutamatergic and lack expression of cholinergic markers such as *cha-1* (choline acetyltransferase) and *unc-17* (acetylcholine transporter), direct cholinergic transmission from TRNs to AVA seems also unlikely. A more plausible model is that the TRN-AVA functions indirectly, requiring additional acetylcholine-releasing interneurons to relay TRN activation to AVA. Based on anatomical connectivity, we propose that AVD and AVE, both of which are cholinergic interneurons that receive strong TRNs inputs fand project to AVA [1,2]. Cooperative activation of AVA, AVD, and AVE—well known premotor interneurons for backward locomotion—could robustly drive nicotine-enhanced reversals. Additional or parallel mechanisms may also contribute, including neuropeptidergic modulation from TRNs [3,4].” We have now incorporated these points in the revised ‘Discussion’ (Lines 493-503). We are grateful for the comment, which prompted us to deepen the mechanistic discussion.

Related to this, Figure 6D is confusing. If the effect is indirect, a solid arrow from TRNs to AVA may be misleading in the circuit diagram.

**Response:**

Thanks. We have replaced the solid arrow with a dashed line in the revised **Fig 6D** to avoid confusion.

2) The finding that nicotine induces reliable reversal behavior tightly coupled to the DMP, especially during the pBoc phase, is intriguing and may reflect a form of interoceptive sensing of body contraction. However, the current data primarily establish a temporal correlation. Have the authors examined nicotine-induced reversal in egl-8, pbo-4, or pbo-5 mutants, which display disrupted pBoc? Additionally, what is observed in nlp-40 or aex-2 mutants, which disrupt DMP rhythmicity? These data could help distinguish whether nicotine-induced reversal depends on DMP timing, contraction, or both, and would strengthen the conclusion that mechanical signals associated with DMP are required for this behavior.

**Response:**

Thank you for your suggestion. In response, we examined nicotine-induced reversal behavior in *egl-8* mutants, which displays disrupted posterior body contraction (pBoc), as well as in *nlp-40* and *aex-2* mutants, which primarily affect expulsion (Exp) [5,6]. As shown in **Fig R1**, nicotine-induced DMP-reversal coupling was significantly impaired in pBoc mutants, but no such effect was observed in Exp mutants. This result suggests that blocking pBoc has the most pronounced impact on nicotine-enhanced motor coupling.

Additionally, we tested *aex-3* mutant, which primarily affect both anterior body contraction (aBoc) and Exp steps [5,7]. This mutant also showed some impact on nicotine-induced abnormal motor coupling compared to wild-type animals, although the effect was less pronounced than in the *egl-8* mutants (**Fig R1**). These observations support the conclusion drawn from temporal correlation analysis: that the nicotine-enhanced coupling of DMP-reversal is mechanistically attributable primarily to body contraction–may reflect a form of interoceptive sensing of body contraction.

**Fig R1. Effect of different DMP mutants on nicotine-enhanced DMP-reversal coupling.** (A) A schematic diagram of *C. elegans* defecation motor program, and. (B) Nicotine-enhanced DMP-reversal coupling events in 10 min was reduced in *egl-8* and *aex-3* mutants, but was not altered in *aex-2* and *nlp-40* mutants. ns, no significance, * *p* < 0.05, ** *p* <0.01, *** *p* < 0.001 by Two-way ANOVA analysis (interaction: F_(4, 81)_ = 15.82, P < 0.0001). Error bars, SEM.

We have integrated these results into the revised manuscript (revised **S2E Fig**), and made corresponding revisions throughout the text (Lines 151-159).

3) The authors report that nicotine exposure increases ACR-16::GFP fluorescence when the protein is expressed under the AVA-specific twk-40s promoter (lines 330-335). However, because this construct uses a heterologous promoter and acr-16 cDNA lacking endogenous introns or regulatory elements, it is difficult to interpret the fluorescence increase as transcriptional upregulation. Indeed, it is somewhat puzzling that this result appears consistent with the knock-in strain, where ACR-16 is expressed under its endogenous regulatory control. This raises questions about whether the observed effect reflects transcriptional regulation, protein stabilization, or some other mechanism. The authors should clarify this point.

**Response:**

Thank you for your thoughtful comment. We acknowledge that using the *twk-40s* promoter to drive ACR-16::GFP may involve non-endogenous regulatory elements. To address this, we also examined a knock-in strain via CRISPR/Cas9 in which ACR-16 is expressed under its native control (ACR-16-AID-Scarlet-spGFP11) in the study (Fig 4A). Both approaches consistently showed increased ACR-16::GFP fluorescence following nicotine exposure, suggesting that the upregulation is unlikely to be driven by transcriptional regulation via endogenous elements. Instead, it likely reflects a post-transcriptional mechanism, which is also observed in rodent brains [8,9].

Another possible mechanism underlying nicotine-induced ACR-16 upregulation is that nicotine acts as a molecular chaperone, promoting the assembly, maturation, and trafficking of ACR-16 subunits. In both heterologous expression systems and native neurons, pharmacological disruption of these processes has been shown to impair nicotine-induced receptor upregulation at the cell surface [10-12]. Similar mechanism could also be applied in vivo to nicotine-induced ACR-16 upregulation in *C. elegans*.

A third possibility is the inhibition of cell-surface ACR-16 receptor turnover by nicotine. In mammalian systems, nicotine has been shown to slow the turnover of surface α4β2 receptors—the major nicotine-sensitive subunits [13]. These findings suggest that receptor accumulation may result from reduced degradation. A similar mechanism could underlie the nicotine-induced upregulation of ACR-16 in *C. elegans* in vivo. Future studies should examine whether blocking ACR-16 turnover in *C. elegans* abolishes the nicotine-enhanced coupling between DMP and reversal.

Although the study does not reveal the specific regulatory mechanisms, we have now expanded the revised ‘Discussion’ (Lines 512-522).

Reviewer #2: The study by Liu and colleagues describes the effects of nicotine exposure on C. elegans behavior, at the neural circuit level. The work revealed that nicotine dramatically increases the coupling between the defecation cycle and reversing during locomotion, by enhancing the expression of a nicotine acetylcholine receptor in the AVA premotor neuron. Intriguingly, this coupling is mediated by the gentle touch mechanosensory neurons, know to respond to external mechanical stimulation, but now suggested to also detect internal movements associated with defecation, thus activating (directly or indirectly) the AVA neurons. The experiments appear well designed and performed, employing both standard methods and challenging techniques. The research is conveyed in a compelling and clear manner. The findings are of great interest to the understanding of how internal and external information may be integrated by the nervous system, and how a high dosage of nicotine may affect neural function. I have a few minor comments, but otherwise highly recommend the publication of this study.

Minor comments:

1.	The term "intact" worms indicating non-exposed controls may be a little confusing. I suggest replacing it with "naïve", "control", or "non-exposed". In any case, after this term is introduced it is never used again, so it could also be removed altogether.

**Response:**

Thank you for your suggestion. We have removed the term "intact" and replaced it with "nicotine-naïve controls (- nic)" and "nicotine-exposed (+ nic)" worms to clearly indicate the experimental conditions in the revised manuscript.

2.	Fig. S1B a statistical test comparing proportions should be applied.

**Response:**

Thanks. We have added statistical analysis of reversal proportion (revised **S1B Fig**) using a Student’s *t* test with *p* < 0.001.

3.	"These high-frequency, prolonged reversals, and high-speed triggered by nicotine resemble nicotine-induced anxious or aversive behaviors observed in mammals (25, 26), suggesting a conserved functional mechanism for nicotine response in C. elegans". I think this statement is too far reaching and circumstantial. I would leave out the second part of this claim, ", suggesting a conserved …".

**Response:**

We have removed the second part of the claim as recommended. Thanks.

4.	Fig 1G instead of temporal dependence, it would be more precise to say dependence on exposure time. Temporal dependence may imply some kind of temporal pattern (e.g., on-off-on).

**Response:**

Thank you for the suggestion. We have replaced "temporal dependence" with "exposure-time dependent" for clarity.

5.	Not quite sure how the coupling proportion was calculated. According to the Methods it sounds like the number of reversals / calcium transients was divided by the number of defecation cycles. However, this does not necessarily imply synchrony. Was there a time window relative to each defecation cycle within which a reversal should have occurred? How was a calcium response vs. no response determined?

**Response:**

Thank you for your question. Indeed, there is a defined time window for the coupling analysis. Based on our data, we found that nearly all DMP-reversal couplings occurred within 5 seconds of DMP initiation (Fig 1E and S2D Fig). In calculating the coupling proportion, we only considered reversals and AVA calcium transients that occurred within this 5-second window following DMP initiation. We have revised the ‘Methods’ section to clarify this approach: “The time window for assessing motor coupling was defined as the 5-second period following DMP initiation, during which reversals and calcium transients were recorded.” (Lines 623-625).

6.	More details about the 2-way ANOVA results should be reported (i.e. the interaction term, the p-value etc.).

**Response:**

Sure, we have added the interaction term and exact p-values of the Two-way ANOVA analysis to the ‘Figure legends’ in the revised manuscript.

7.	In Fig. 2E (bottom) it is worth labeling the separate and overlayed channels, just for clarity.

**Response:**

Thanks. We have labeled the channels for clarity in revised **Fig 2E**.

8.	Fig. S4 is supposed to show normal nicotine behavioral responses in the GCaMP strain (line 286), but shows instead the effects of the acr-16 mutation (even though these were discussed earlier).

**Response:**

Thank you for your comment. We intended to show that the wild-type AVA::GCaMP transgenic strain exhibits nicotine dependent behavioral responses similar to those observed in N2 animals. We apologize for the unclear phrasing. We have revised the sentence to: " These AVA::GCaMP transgenic animals responded to nicotine similarly to wild-type N2 worms in terms of reversal frequency, duration, and locomotion speed (wild type, S4 Fig)" (Lines 266-268). Meanwhile, we have also revised the annotation of **S4 Fig** to indicate the transgenic background, so as to avoid any misunderstanding.

9.	Regarding the acr-16:GFP strain, can you please provide more information in the Methods about how it was constructed, including relevant references?

**Response:**

Sure, P*acr-16*::GFP reporter construct was generated using the Gateway recombination system (Invitrogen) in combination with In-Fusion cloning. To make the entry vectors, a 3.5 kb of sequence upstream of the *acr-16* gene were PCR amplified from *C. elegans* N2 genomic DNA using gene-specific promoter primers containing attB1 and attB2 sequences. The gene-specific sequences for P*acr-16* are as follows:

Forward: 5′-AGTTGCTCCCTCGAGACATTCACAACATATACGGTG-3′

Reverse: 5′-CTTGCACGACTAGTGGAGTGAATGTACATCCG-3′

The amplified promoter fragment was inserted into a linearized Gateway entry vector (Entry Clone A) using In-Fusion cloning, generating an entry clone containing the *acr-16* promoter flanked by attL1 and attL2 recombination sites.

An LR recombination reaction was subsequently performed between the entry clone and a Gateway destination vector harboring GFP and the *unc-54*-3'UTR, resulting in the final expression construct, P*acr-16*::GFP::*unc-54*-3'UTR. We have incorporated them into the revised ‘Methods’ section. (Lines 554-567)

10.	I suggest to replace "neurotransmitter" with transmitter or signal when referring to H+ (line 377).

**Response:**

Sure, we have replaced the "neurotransmitter" with "transmitter".

11.	Fig. 3C unclear how the calcium response was quantified and why does it have discrete values.

**Response:**

Thank you for pointing this out. We have clarified the calcium response quantification method as follows:

“A calcium response was defined as an increase in fluorescence intensity of ≥ 10% that occurred with a reversal behavior immediately following the pBoc phase of the DMP. Each such event was counted as one calcium response. We then calculated the calcium response percentage by dividing the number of these response events by five—the total number of DMP cycles analyzed per animal. This quantification yields discrete values (e.g., 0, 20%, 40%, ..., 100%) corresponding to the number of DMP cycles that showed a coupled calcium response. To minimize signal drift and motion artifacts, we limited our analysis to five consecutive DMP cycles per worm.”

We have now incorporated this clarification into the ‘Methods’ sections of the revised manuscript. (Lines 680-688)

12.	In Fig. 5A the coupling is shown for the various mutants. Is the DMP cycle the same across mutants (especially TRN-related mutations)? For example, it has been shown that mec-4 have an altered the reversing frequency (both on and off food). How does this affect the coupling?

**Response:**

Thanks. We conducted a detailed analysis of the DMP cycle in wild-type N2 and several TRN-related mutants, including *mec-2*, *mec-4*, and *mec-6*, and found no significant differences in the DMP cycle across these mutants (**Fig R2A**). Additionally, we tested the *mec-4(e1611)* loss-of-function mutant in our study and observed no significant changes in the reversing frequency, both on food and off food (**Fig R2B**), consistent the report in in this same mutant allele that spontaneous reversing rate is not affected in the absence of food [14]. Regarding your mention of *mec-4* affecting the reversing frequency is likely associated with a different allele of *mec-4(u253)* [14]. To avoid potential locomotion defects interfering with the analysis of motor coupling, we used *mec-4(e1611)* in this study.

**Fig R2. DMP cycle and reversing frequency are normal in TRN-related mutations.** (A) DMP cycle in wild-type N2 and *mec-2(e75)*, *mec-4(e1611)*, and *mec-6(e1342)* mutants. (B) Quantification of the spontaneous reversing rate (# of reversals per 3 minutes) in the presence (On food) and in the absence (Off food) of food, for normal worms (N2) and *mec-4(e1611)* mutants. ns, not significant by Two-way ANOVA analysis (interaction: F_(3, 712)_ = 1.238, P = 0.2948 for (A); F_(1, 36)_ = 0.08663, P = 0.7702 for (B)). Error bars, SEM.

13.	In Fig. 6D,E TRN-triggered AVA EPSPs are presented. In the Results this is inferred as TRN-AVA synaptic strength. However, as pointed out in the Discussion, this may likely be an indirect pathway, and so the phrasing should be changed in the Results and the Introduction.

**Response:**

Thank you for your insightful comment. We have revised the phrasing in both the ‘Introduction, Results, and Figure legends’ to replace "synaptic strength" with "signal strength" or "neural activity", as highlighted in the ‘Discussion’.

14.	The AVA rescue promoter (Ptwk-40) is not specific. When was the intersection promotor mentioned in the Methods used?

**Response:**

Thank you for pointing this out. The short *twk-40* promoter (*Ptwk-40s*) is indeed not specific to AVA, as it drives stable expression in both AVA and AVB neurons. However, our data indicate that ACR-16 expression in AVB alone does not rescue the functional deficit observed in *acr-16* mutants (Fig 2F). Thus, the rescue effect observed with P*twk-40s* likely reflects ACR-16 function in AVA.

Regarding the intersectional promoter, it was used in the result section “Nicotine upregulates functional expression of ACR-16.” As now clarified in the revised ‘Results and Methods’, we inserted spGFP11 into the endogenous *acr-16* locus and expressed spGFP1-10 specifically in AVA using a Cre-loxP–based promoter combination (Methods). (Lines 311-317)

15.	How was the eye lash touch assay performed? Was it on or off food?

**Response:**

Thank you for your question. The eyelash touch assay was performed on food, consistent with the conditions used for locomotion analysis. We have added this detail to the revised ‘Methods’ section for clarity (Line 631).

16.	The manuscript could benefit from overall further English editing.

**Response:**

We thank your valuable suggestion. We have carefully revised the entire manuscript with a focus on refining language expression, ensuring grammatical accuracy, and enhancing the fluency of scientific descriptions. Additionally, we have sought assistance from a native English-speaking colleague with expertise in neuroscience to proofread the revised manuscript. We believe these revisions have significantly improved the quality of the manuscript, and we appreciate your guidance in this regard.

Reviewer #3 [Vincent O'Connor]: The submission is an interesting study that provides a plausible mechanistic explanation for a nicotine induced behavioural response on motility and defecation. There is a detailed unpicking of the previously described loose coupling between these grosser behaviours. The text draws a lot of overviews from the mammalian literature subserved by the addictive issues of nicotine. I found this aspect a tad inflated and personally not required to support the value of the study. In addition, I think the grandiose against exteroceptive and interoceptive cueing of organismal behaviour difficult to follow. If the authors wish to pursue this tack, they should better justify it and address some of the clarifications that their study design raises. This is an interesting study, and one must recognize its technical merits.

**Response:**

Dear Dr. O'Connor,

Thank you very much for your thoughtful and constructive comments, as well as for recognizing the novelty and technical merits of our study. We appreciate your feedback regarding the tone and clarity of the manuscript.

In response, we have accepted your insightful suggestion and removed the discussion on the neuroanatomical overlaps between interoception and exteroception in mammals. We have also revised the relevant paragraphs to streamline the interpretation. Additionally, we have added mechanistic implications of our findings, particularly focusing on the potential involvement of additional acetylcholine-releasing neurons in mediating TRN-induced AVA activation (Lines 493–503) and the possible in vivo signaling mechanisms underlying nicotine-induced upregulation of ACR-16 (Lines 512–522).

We hope these revisions will reduce potentially inflated descriptions and enhance the discussion of the mechanisms underlying our findings. Thank you again for your valuable suggestions.

Major point

The paper reads like a pharmacological dosing regime that sets up behavioural plasticity. Give this better detailing or clarity about the experiment is needed.

The methods imply that OP50 dosed with nicotine is used to set up the behavioural paradigm. My reading would be that 100 microliters of a stock is added to a 3 ml agar plate. This suggests 30mM nicotine are added to the bacteria. If this is the case this would be a dose that is strongly antimicrobial. An alternative interpretation of the methods is that 1mM is added to the OP50 and after an ill-defined diffusion into the agar reaches approx. 30 microM dosing. As the authors introduction nicely points out nicotine does many things across the possible dose ranges.

**Response:**

Thank you. We now clarify the description of the nicotine plate preparation protocol as follows: “4.86 μL of nicotine (Shanghai PureOne Biotechnology, L-Nicotine, HPLC purity ≥ 98%) was added into 1 mL of an *E. coli* OP50 suspension to create a 30 mM nicotine-OP50 mixture after 1 minute vortexed. 100 μL of this mixture was then dropped and spread onto the surface of a standard 3 mL NGM agar plate, and the NGM was shaken to ensure that the mixed solution evenly covered the entire surface. These nicotine plates were left at 22 °C for 16 hours to allow diffusion of nicotine into the agar and OP50 bacterial lawn formation. The final nicotine concentration in these plates is approximately 1 mM.” (Lines 604-612)

In response to your concern regarding potential antimicrobial effects of high nicotine concentrations on OP50, we performed the following additional experiments：

(1) OP50 growth quantification using GFP-expressing bacteria:

We directly compared the growth of *E. coli* OP50 lawns with and without nicotine. To facilitate quantification, we used a GFP-expressing OP50 strain and monitored bacterial growth over 24 hours; this strain exhibits a growth rate identical to that of the unlabeled wild-type OP50.

Under this condition, we observed no significant difference in bacterial growth at the edges of the lawns during the first 12 hours (quantified by GFP intensity) with and without nicotine-exposure (**Fig R3A, B**). At 24 hours, there was a slight (~10%) reduction in fluorescence intensity under the nicotine condition. However, the inner regions of the lawns showed no significant difference between the two conditions (**Fig R3C**). These results suggest that nicotine does not substantially inhibit OP50 growth over the relevant experimental timescale, and are consistent with the approach of pre-equilibrating nicotine into solid NGM [15].

**Fig R3. *E. coli* OP50 bacterial lawn growth is approximately normal with 1 mM nicotine.** (A) OP50 bacteria expressing GFP (OP50::GFP) were used to analyze the effect of nicotine. *Left*: Schematic diagram of the NGM plate with OP50::GFP. *Right*: OP50::GFP fluorescence intensity at different time points without (- nic) and with (+ nic) 1 mM nicotine. The dashed boxes represent the fluorescence sampling locations: 1. White box: marginal region of the bacterial lawn; 2. Green box: inner region of the bacterial lawn. (B, C) Quantification of OP50::GFP fluorescence intensity at different sampling locations. ns, not significant; * *p* < 0.05; ** *p* < 0.01 by Two-way ANOVA analysis (interaction: F_(5, 108)_ = 2.974, P = 0.0149 for (B); F_(5, 108)_ = 0.2127, P = 0.9565 for (C)). Error bars, SEM.

(2) Comparison to established nicotine plate preparation method:

To further ascertain the potential antimicrobial effect of 1 mM nicotine on OP50, we also compared the method used in this study—where nicotine was mixed with OP50 prior to plating—with that of Kudelska *et al.* [15], who pre-equilibrated nicotine into solid NGM 12 hours in advance. After 24 hours, the growth of OP50::GFP on NGM plates prepared by both methods was identical, albeit slightly reduced compared to plates without nicotine (**Fig R4A, B**). Additionally, 1 mM nicotine NGM plates prepared using either method induced nearly 100% DMP-reversal coupling (**Fig R4C**).

**Fig R4. Different nicotine preparation approaches induce the same coupling proportion.** (A) *Left*: Schematic diagram of the NGM plate with OP50::GFP. *Right*: OP50::GFP fluorescence intensity under different nicotine diffusion approaches. - nic: NGM plates under standard culture condition without nicotine. + nic (NGM): Nicotine pre-diffused into solid NGM for 12 hours prior to the addition of OP50::GFP. + nic (OP50): Nicotine mixed with OP50::GFP and co-applied to NGM plates. (B) Quantification of OP50::GFP fluorescence intensity at 24 hours post-plating under different conditions. OP50 bacterial lawn growth appears normal, with no significant difference observed between the two nicotine treatment conditions. (C) Both the + nic (NGM) and + nic (OP50) conditions induced comparable levels of enhanced DMP-reversal coupling proportions. ns, not significant by Students’ *t* test. Error bars, SEM.

(3) Nicotine diffusion analogy experiment:

Above experimental results suggest that the 1 mM nicotine is not toxic to bacterial growth, likely due to its rapid diffusion into the agar. To further assess the diffusion rate of nicotine on NGM plates, we attempted to visualize its spread over time using Dragendorff’s reagent. However, due to the complexity of the NGM medium and other unknown factors, Dragendorff’s reagent failed to effectively develop color for nicotine in NGM and could not distinguish the dynamic diffusion of nicotine.

To gain a more intuitive understanding of nicotine diffusion, we then turned to fluorescein sodium as a proxy to simulate nicotine diffusion. This choice was based on several considerations: (1) fluorescein sodium fluoresces under appropriate light, allowing real-time visualization and quantification; (2) it shares similar solubility and polarity characteristics with nicotine, making it compatible with the NGM environment; and (3) its molecular weight is slightly larger than that of nicotine (fluorescein sodium: 376.27; nicotine: 162.23), so theoretically, it may diffuse a bit more slowly than nicotine. Although their dissolution kinetics may not be identical, we reasoned that observing the diffusion behavior of fluorescein sodium could serve as a reference for nicotine’s diffusion dynamics.

As shown in **Fig. R5A**, when co-seeded with OP50 bacteria, fluorescein sodium diffused evenly across the NGM plate within approximately 30 minutes. This distributed intensity was comparable to that observed when fluorescein sodium was pre-mixed into the NGM prior to plate pouring (**Fig. R5B, C**).

It should be noted that this is merely an attempted analogous observation and cannot illustrate the diffusion efficiency of nicotine in NGM.

**Fig R5. Fluorescein sodium diffuses rapidly in NGM plates.** (A) *Left*: Fluorescein sodium (1 mM) was pre-mixed into NGM before pouring, resulting in uniform fluorescence across all agar layers. *Right*: Fluorescein sodium was co-seeded with OP50 on the surface of NGM plates, and fluorescence distribution was imaged at different time points post-seeding. (B) Schematic comparison of fluorescence distribution under the two experimental conditions. (C) Quantification of fluorescein sodium fluorescence intensity across different layers of the NGM (top, middle, bottom) over time. The results indicate that fluorescein sodium diffuses throughout the agar and reaches uniform distribution within approximately 30 minutes.

Given the 3-hr incubation period it would be important that the authors make clear statements about when animals were evaluated relative to the on-food dosing. Nicotine is charged and would not wash out quickly once accumulated in the worm. Do the authors think they are assaying in the absence on intra organismal nicotine. If this is not the case, I would be cautious about invoking interoceptive effects of nicotine when you are simply studying the impact of circulating levels of intoxicating nicotine.

**Response:**

Thank you for this insightful comment, which allows us to clarify our interpretation of nicotine’s interoceptive effects in the context of possible intra-organismal residues. In this study, animals were directly transferred to standard NGM plates with a normal OP50 lawn after 3 hours of incubation on nicotine-containing plates. A brief 3-minute adaptation period was set before evaluating motor coupling behavior [16].

We agree that complete elimination of residual nicotine following exposure is technically challenging, and acknowledge that some nicotine may remain within the organism during the assay. However, we believe this does not conflict with our focus on interoception. By “interoceptive effects,” we refer to the internal sensing and neural processing of nicotine-related signals—whether from acute exposure or residual presence within the body.

Interoception, by definition, involves the monitoring of internal physiological states. In this context, residual nicotine constitutes a component of the organism’s internal milieu and may contribute to the interoceptive signals being processed. Supporting this, we observed that nicotine-enhanced coupling was reversible: when animals were transferred to a nicotine-free environment, the DMP-reversal coupling gradually decreased, returning to baseline (~20%) with a half-life of ~1.2 hours (Fig 1H). This gradual decline is consistent with the internal sensing and processing of nicotine clearance.

In summary, while residual intra-organismal nicotine may remain during the assay, we propose that it is precisely this internal presence—and the organism’s ability to sense and respond to it—that constitutes the interoceptive effect.

The study has some interesting controls, but it does not report on how sustained the nicotine induced intoxication is on defecation program. It is interesting that the onset of effects is fast 15 mins. I would also caution that this can put gustatory cueing as a potential explanation. Given the authors prowess in behavioural recording it would be interesting to know about the nature of the bacterial lawns laced with nicotine, the behaviour of the worms while on nicotine (body length would be an obvious measure) and whether there is aversion when place on the nicotine laced lawns.

**Response:**

Thanks. As shown in S2A-C Fig, we observed that a 3-hour exposure to nicotine did not significantly alter the endogenous defecation motor program cycle, suggesting that nicotine does not disrupt the timing of the defecation rhythm.

To address your concern regarding potential gustatory cueing, we tested primary gustatory mutants, including *che-1*—a zinc-finger transcription factor exclusively expressed in the ASE neuron pair that mediates gustation [17,18], as well as *tax-2* and *tax-4*, which encode a gustatory neuron-specific heteromeric CNG channel complex [19,20]. All mutants exhibited wild-type like DMP-reversal coupling under same experimental conditions (**Fig. R6**), suggesting that gustatory sensation is not required for nicotine-induced coupling changes.

**Fig R6. DMP-reversal coupling in gustatory mutants.** Quantification of coupling proportion in wild-type N2, *che-1(ot124), tax-2(p691)* and *tax-4(sk11)* mutants, *** *p* < 0.001, by Two-way ANOVA analysis (interaction: F_(3, 72)_ = 0.8182, P = 0.4880).

Regarding the nature of the nicotine-laced bacterial lawns, we assessed OP50 growth and found that 1 mM nicotine did not significantly inhibit lawn formation (**Fig. R3**). As you suggested, we also measured worm body length at multiple time points from 15 minutes to 3 hours after 1 mM nicotine exposure and observed no significant changes (**Fig. R7**), indicating no obvious nicotine-induced tonic contraction or body shrinkage.

**Fig R7. Nicotine-exposure does not change body length.** (A) Representative body lengths after different durations of nicotine-exposure. (B) Quantification of body lengths under the corresponding experimental conditions. ns, not significant by Students’ *t* test. Error bars, SEM.

Finally, we did observe behavioral aversion when placing intact worms onto nicotine-containing lawns: animals exhibited immediate increases in locomotor activity and frequent reversals, consistent with an aversive response, similar to that previously reported by Feng *et al*. [16].

While these behavioral observations and mechanistic considerations are indeed intriguing, the present study focuses primarily on the behavioral mechanisms of motor coupling. Thus, we have not elaborated on all details herein, and we aim to further refine the analysis of distinct behavioral patterns and their underlying mechanisms in future studies.

The description of the behavioural paradigm is clear. Intoxication on food followed by an ill-defined experimental recovery on agar arenas in the absence of food.

1.	If the nicotine compromises the food lawns, there will be a distinct food quality.

2.	If the high doses of nicotine impacts feeding behaviour. It will at the higher dose range: there will be a food intake difference.

3.	If either of these pertain the interoceptive cueing (food levels) will contribute to or overlap with confounding nicotine effects.

In the experiments it would help if the authors were clear about whether DHBE is present during the behavioural intoxication or present when the cellular (imaging or electrophysiology assays are being performed. Again, the time between removal from intoxication 3 hrs and experimental measurement would always be informative.

In essence setting up the paradigm would help the reader better ascertain if the nicotine has driven a behavioural state or the behavioural state is being measured in the presence of pharmacologically relevant drug.

Minor points.

Was eat-2 tested in the screen for molecular determinants. It would be a useful check for some of the issues raised above.

Ric-7 would have been an interesting/useful addition to this data.

I agree that interpreting the data of the body wall muscle mutants is challenging but I think a preconditioning with levamisole would be especially useful as the body wall muscle contraction that nicotine might induce would be controlled for and add mechanistic clarity. This point is raised by the authors nice data highlighting a mechanosensitive cueing to the behavioural response.

useful ref: Invert Neurosci. 2018 Nov 7;18(4):14. doi: 10.1007/s10158-018-0219-1.

**Response:**

Thank you for your insightful comments and the recommended references. To address your questions, we will respond to each of the key points as follows:

(1) Food quality and intake under nicotine exposure:

As shown in Fig. R3 and Fig. R4, 1 mM nicotine does not substantially affect food quality. Following your suggestion, we further examined whether nicotine’s effect on motor coupling is related to food intake by testing *eat-2(ad1116)* mutants, which have defective feeding due to impaired pharyngeal pumping. Under the same experimental conditions, *eat-2(ad1116)* mutants exhibited wild-type-like nicotine-induced abnormal DMP-reversal coupling (**Fig. R8**), despite their reduced food intake. These results indicate that reduced feeding per se does not account for the coupling phenotype and suggest that nicotine’s effect on coupling is not simply mediated by altered food intake.

**Fig R8. Nicotine-enhanced motor coupling in *eat-2* mutant.** Nicotine enhanced coupling proportion was not altered in *eat-2(ad1116)* mutant. ns, not significant, *** *p* < 0.001, by Two-way ANOVA analysis (interaction: F_(1, 36)_ = 0.2169, P = 0.6442). Error bars, SEM.

(2) Clarification of pharmacological conditions (DHβE presence and timing):

We appreciate your request for clarity regarding DHβE usage. In all experiments where pharmacological inhibition of ACR-16 was tested (Fig 2A, S3, S5, S6), DHβE was present during nicotine exposure. In all other experiments, DHβE was not included. We have clarified this point in the ‘Methods’ section.

(3) Timing of behavioral measurement after intoxication:

Behavioral assessments were conducted immediately after 3 hours of nicotine exposure, without a washout period. We agree that this likely reflects a persistent pharmacological state rather than a recovered baseline. We have added a statement in the revised ‘Discussion’ noting that the measured behavioral state likely results from continued nicotine presence.

(4) *ric-7* analysis:

Following your suggestion, we tested *ric-7* mutants, which have defective mitochondrial transport and broad neurotransmission impairments, including in mechanosensation [21]. *ric-7(nu447)* mutants showed markedly diminished reversal speed upon gentle touch compared to wild-type animals (**Fig. R9A**), indicating impaired mechanosensory function. Under our experimental conditions, *ric-7(nu447)* mutants also exhibited a modest reduction in nicotine-induced DMP-reversal coupling (**Fig. R9B**). Given that *ric-7* disrupts touch-evoked mechanosensation, this result supports the notion that mechanosensory input from TRNs mediates the nicotine-induced coupling phenotype.

**Fig R9. Nicotine-induced DMP-reversal coupling in *ric-7* mutant.** (A) *Top*, schematic illustrating gentle touch-evoked reversal. *Bottom left*, raster plots showing individual speed responses of animals of the indicated genotypes to gentle anterior touch without nicotine exposure (n = 15 animals per group). Arrows indicate the timing of the touch stimulus. *Bottom right,* average speed traces reveal a significant reduction in reversal speed following gentle touch in *ric-7* mutants. (B) Nicotine-enhanced DMP-reversal coupling was slightly reduced in *ric-7* mutants, * *p* < 0.05, *** *p* < 0.001, by Two-way ANOVA analysis (interaction: F_(1, 51)_ = 1.176, P = 0.2833). # *p* < 0.05, by Students’ *t* test. Error bars, SEM.

(5) Levamisole preconditioning experiment:

Following your suggestion, we performed levamisole preconditioning to test whether paralyzing body wall muscles could disrupt nicotine-enhanced motor coupling. We first tested 1 mM levamisole, but this concentration fully paralyzed the worms and completely abolished both DMP and locomotion. We then applied 0.1 mM levamisole for 3 minutes before behavioral testing. At this concentration, levamisole caused marked body contraction (notably in the head), uncoordinated movement and prolonged DMP cycles (**Fig. R10A**). However, it did not affect nicotine-enhanced DMP-reversal coupling (**Fig. R10B**). These results indicate that partial activation of body wall muscles by levamisole alters DMP cycles but does not disrupt nicotine-enhanced motor coupling, suggesting that the coupling is not simply due to nicotine-induced muscle hypertonia.

**Fig R10. Levamisole prolongs the DMP cycle but does not affect nicotine-induced DMP-reversal coupling.** (A) Preconditioning with 0.1 mM levamisole for 3 minutes significantly prolonged the DMP cycle, both in the absence and presence of nicotine (1 mM). (B) Despite extending the DMP cycle, levamisole preconditioning did not affect the nicotine-enhanced coupling between DMP and reversal. ns, not significant; *** *p* < 0.001; by Two-way ANOVA analysis (interaction: F_(1, 356)_ = 0.1414, P = 0.7072 for (A); F_(1, 32)_ = 0.5517, P = 0.4630 for (B)).

We thank you for these valuable suggestions. The relevant results have been incorporated into the revised manuscript, the experimental procedures have been clarified, and appropriate literature has been cited. We appreciate that these refinements have strengthened our paper and enhanced the clarity and interpretability of our findings.

**Reference**

1.	Chalfie M, Sulston JE, White JG, Southgate E, Thomson JN, Brenner S. The neural circuit for touch sensitivity in Caenorhabditis elegans. J Neurosci. 1985;5(4):956-64. Epub 1985/04/01. doi: 10.1523/JNEUROSCI.05-04-00956.1985. PubMed PMID: 3981252; PubMed Central PMCID: PMCPMC6565008.

2.	Cook SJ, Jarrell TA, Brittin CA, Wang Y, Bloniarz AE, Yakovlev MA, et al. Whole-animal connectomes of both Caenorhabditis elegans sexes. Nature. 2019;571(7763):63-71.

3.	Taylor SR, Santpere G, Weinreb A, Barrett A, Reilly MB, Xu C, et al. Molecular topography of an entire nervous system. Cell. 2021;184(16):4329-47 e23. Epub 2021/07/09. doi: 10.1016/j.cell.2021.06.023. PubMed PMID: 34237253; PubMed Central PMCID: PMCPMC8710130.

4.	Sieburth D, Ch'ng Q, Dybbs M, Tavazoie M, Kennedy S, Wang D, et al. Systematic analysis of genes required for synapse structure and function. Nature. 2005;436(7050):510-7. Epub 2005/07/29. doi: 10.1038/nature03809. PubMed PMID: 16049479.

5.	Thomas JH. Genetic analysis of defecation in Caenorhabditis elegans. Genetics. 1990;124(4):855-72. Epub 1990/04/01. doi: 10.1093/genetics/124.4.855. PubMed PMID: 2323555; PubMed Central PMCID: PMCPMC1203977.

6.	Wang H, Girskis K, Janssen T, Chan JP, Dasgupta K, Knowles JA, et al. Neuropeptide secreted from a pacemaker activates neurons to control a rhythmic behavior. Curr Biol. 2013;23(9):746-54. Epub 2013/04/16. doi: 10.1016/j.cub.2013.03.049. PubMed PMID: 23583549; PubMed Central PMCID: PMCPMC3651789.

7.	Iwasaki K, Toyonaga R. The Rab3 GDP/GTP exchange factor homolog AEX-3 has a dual function in synaptic transmission. Embo J. 2000;19(17):4806-16. doi: DOI 10.1093/emboj/19.17.4806. PubMed PMID: WOS:000089275600036.

8.	Marks MJ, Pauly JR, Gross SD, Deneris ES, Hermans-Borgmeyer I, Heinemann SF, et al. Nicotine binding and nicotinic receptor subunit RNA after chronic nicotine treatment. Journal of Neuroscience. 1992;12(7):2765-84.

9.	Bencherif M, Fowler K, Lukas RJ, Lippiello PM. Mechanisms of up-regulation of neuronal nicotinic acetylcholine receptors in clonal cell lines and primary cultures of fetal rat brain. The Journal of pharmacology and experimental therapeutics. 1995;275(2):987-94.

10.	Nashmi R, Dickinson ME, McKinney S, Jareb M, Labarca C, Fraser SE, et al. Assembly of α4β2 nicotinic acetylcholine receptors assessed with functional fluorescently labeled subunits:: Effects of localization, trafficking, and nicotine-induced upregulation in clonal mammalian cells and in cultured midbrain neurons. Journal of Neuroscience. 2003;23(37):11554-67. PubMed PMID: WOS:000187397400006.

11.	Sallette J, Pons S, Devillers-Thiery A, Soudant M, de Carvalho LP, Changeux JP, et al. Nicotine upregulates its own receptors through enhanced intracellular maturation. Neuron. 2005;46(4):595-607. doi: DOI 10.1016/j.neuron.2005.03.029. PubMed PMID: WOS:000229294300011.

12.	Kuryatov A, Luo J, Cooper J, Lindstrom J. Nicotine acts as a pharmacological chaperone to up-regulate human α4β2 acetylcholine receptors. Mol Pharmacol. 2005;68(6):1839-51. doi: DOI 10.1124/mol.105.012419. PubMed PMID: WOS:000233340200035.

13.	Peng X, Gerzanich V, Anand R, Whiting PJ, Lindstrom J. Nicotine-induced increase in neuronal nicotinic receptors results from a decrease in the rate of receptor turnover. Mol Pharmacol. 1994;46(3):523-30.

14.	Staum M, Abraham AC, Arbid R, Birari VS, Dominitz M, Rabinowitch I. Behavioral adjustment of C. elegans to mechanosensory loss requires intact mechanosensory neurons. PLoS Biol. 2024;22(7):e3002729. Epub 2024/07/18. doi: 10.1371/journal.pbio.3002729. PubMed PMID: 39024405; PubMed Central PMCID: PMCPMC11288434.

15.	Kudelska M, Lewis A, Ng C, Doyle D, Holden-Dye L, O’Connor V, et al. Investigation of feeding behaviour in C. elegans reveals distinct pharmacological and antibacterial effects of nicotine. Invertebrate Neuroscience. 2018;18(4):14.

16.	Feng Z, Li W, Ward A, Piggott BJ, Larkspur ER, Sternberg PW, et al. A C. elegans model of nicotine-dependent behavior: regulation by TRP-family channels. Cell. 2006;127(3):621-33. Epub 2006/11/04. doi: 10.1016/j.cell.2006.09.035. PubMed PMID: 17081982; PubMed Central PMCID: PMCPMC2859215.

17.	Etchberger JF, Lorch A, Sleumer MC, Zapf R, Jones SJ, Marra MA, et al. The molecular signature and cis-regulatory architecture of a C. elegans gustatory neuron. Genes Dev. 2007;21(13):1653-74. Epub 2007/07/04. doi: 10.1101/gad.1560107. PubMed PMID: 17606643; PubMed Central PMCID: PMCPMC1899474.

18.	Uchida O, Nakano H, Koga M, Ohshima Y. The C. elegans che-1 gene encodes a zinc finger transcription factor required for specification of the ASE chemosensory neurons. Development. 2003;130(7):1215-24. Epub 2003/02/18. doi: 10.1242/dev.00341. PubMed PMID: 12588839.

19.	Coburn CM, Bargmann CI. A putative cyclic nucleotide–gated channel is required for sensory development and function in C. elegans. Neuron. 1996;17(4):695-706.

20.	Komatsu H, Mori I, Rhee JS, Akaike N, Ohshima Y. Mutations in a cyclic nucleotide-gated channel lead to abnormal thermosensation and chemosensation in C. elegans. Neuron. 1996;17(4):707-18. Epub 1996/10/01. doi: 10.1016/s0896-6273(00)80202-0. PubMed PMID: 8893027.

21.	Rawson RL, Yam L, Weimer RM, Bend EG, Hartwieg E, Horvitz HR, et al. Axons degenerate in the absence of mitochondria in C. elegans. Curr Biol. 2014;24(7):760-5. Epub 2014/03/19. doi: 10.1016/j.cub.2014.02.025. PubMed PMID: 24631238; PubMed Central PMCID: PMCPMC4018749.
