# Supplementary material for: Nicotine induces abnormal motor coupling through sensitization of a mechanosensory circuit in Caenorhabditis elegans
Source: PLoS Biol. 2025 Oct 3;23(10):e3003423. doi: 10.1371/journal.pbio.3003423 (PMC12507281; doi:10.1371/journal.pbio.3003423)
Supplement: Attachment — Submitted filename: Reponse letter_R2 (PBIOLOGY-D-25-01396).docx [file pbio.3003423.s017.docx]

REVIEWS:

Reviewer #1: I appreciate the new data and extensive text revisions. The revised manuscript is stronger, with several additions that enhance the study. For example, the authors added new controls on nicotine diffusion and food quality, included egl-8, aex-3, aex-2, and nlp-40 mutants to dissect the role of DMP phases, and provided a clearer explanation of methods such as how DMP-reversal coupling was measured, together with appropriate statistical analysis. These changes substantially improve the rigor of the work, even though the precise circuit connection between TRNs and AVA remains unresolved. With these improvements in place, I outline below a few remaining concerns.

**Response:**

We are very grateful for your positive assessment and constructive feedback. We truly appreciate the recognition of our efforts to improve the manuscript.

1) ACR-16 regulation

In response to my prior comment regarding interpretation of the AVA reporter (Ptwk-40s::acr-16cDNA::gfp), the authors conclude that ACR-16 upregulation is mediated by a post-transcriptional mechanism (lines 513-523). However, this is difficult to reconcile with the data in Figures S5A and 5B. As described in lines 307-311, the transgenic strain expresses functional ACR-16 cDNA tagged with SL2d::GFP in AVA neurons. Because the SL2 trans-splicing element should produce GFP and ACR-16 as separate proteins, this construct would primarily reflect transcriptional changes rather than post-transcriptional regulation. It seems unlikely that GFP and ACR-16, expressed separately, would be subject to the same post-transcriptional control. Since ACR-16 upregulation is central to the proposed mechanism of nicotine sensitization of AVA activation and reversal behavior, this point requires clarification.

**Response:**

Thank you for pointing out this important concern. Our interpretation of a post-transcriptional mechanism was based on the observation that heterologous promoter (P*twk-40s*)-driven *acr-16* cDNA exhibited significant upregulation upon nicotine exposure, suggesting that nicotine-induced ACR-16 upregulation does not necessarily require the endogenous *acr-16* promoter. In other words, nicotine may not act directly on the endogenous *acr-16* promoter region, which is typically central to transcriptional regulation. That said, we agree that we cannot exclude the possibility that the heterologous promoter itself may also be responsive to nicotine-evoked transcriptional regulation. To avoid over-interpretation, we have revised the text to present this point more cautiously. (Lines 528-530)

2) Discussion of "interoceptive effects" versus residual nicotine

While I appreciate the authors' clarification that they define "interoceptive effects" as internal sensing and neural processing of nicotine-related signals, I agree with Dr. O'Connor that a "circulating intoxicant" is not necessarily equivalent to a "sensed internal state." Whether lingering nicotine represents interoceptive sensing or simply residual intoxication remains only partially resolved. The current justification relies on a broad interpretation of interoception that may not align with stricter definitions. In other words, the mere presence of nicotine does not constitute an interoceptive process unless specific internal sensors are engaged, rather than passive pharmacological effects. That said, both interpretations are interesting. To avoid confusion for readers, I suggest the authors include a clearer discussion distinguishing between residual pharmacological effects and bona fide interoceptive processes.

**Response:**

Thank you for discussing this point again. In our study, we have used a broad definition of interoception to describe the internal sensing and processing of nicotine-related signals. Indeed, we cannot exclude the possibility that residual nicotine remaining after exposure may directly influence behavior as a lingering intoxicant. This alternative explanation highlights an important nuance: while the presence of residual nicotine reflects a pharmacological state, it becomes an interoceptive process only when the organism actively senses and processes this internal condition. While the precise contribution of pharmacological versus sensor-mediated mechanisms remains unresolved, our findings emphasize a potential continuum between residual drug effects and bona fide interoceptive sensing.

We thank you for this suggestion and now explicitly distinguish residual pharmacological effects from bona fide interoceptive processes in the Discussion. (Lines 518-525)

3) Minor comments:

Line 140: Change "These results suggests…" to "These results suggest…".

Line 163: Change "This alignment allowed to precisely synchronize…" to "This alignment allowed us to precisely synchronize…".

Line 180: Change "nicotine act…" to "nicotine acts…".

Line 394: Change "To exam this…" to "To examine this…".

Line 423: Remove "of" and change to "nicotine enhances the signal strength from TRNs to AVA."

Line 429: Change "depending" to "depend".

Line 461: Change "exhibiting" to "exhibit".

Line 468: Change "This dues to…" to "This is due to…".

Line 501: Correct "fand" to "and".

**Response:**

We thank you for carefully checking the manuscript and pointing out these grammatical errors. We have corrected all the mentioned typos and grammatical issues in the revised version (Lines 141, 164, 181, 399, 428, 434, 466, 473, and 506). In addition, we have also re-checked the manuscript thoroughly to correct similar errors.

Reviewer #2: I appreciate the thorough revision and responses.

My comments have been almost completely addressed. I have just two very minor follow-up comments:

Comment 2: For S1B, a t-test is not a good test for comparing proportions. Rather, a Z test, for example, would much more appropriate.

**Response:**

Thanks. We have additionally performed a Z test for proportions. The results were highly consistent with our original analysis (p < 0.001). We have revised the Methods and legend of Fig. S1B accordingly.

Comment 12: The extra data provided on the mechanosensory mutants and the clarification regarding e1611 is very informative. I recommend adding this to the manuscript.

**Response:**

Thank you for your suggestion. We have incorporated the extra data on mechanosensory mutants, including analysis of *mec-4(e1611)*, into the revised manuscript as suggested (see S7 Fig and Lines 373-377).

Reviewer #3 [Vincent O'Connor]: I am more than impressed with the effort and quality embedded within the revised paper. This provides excellent reassurance and I hope important clarification and insight around dosing.

I leave it to the authors to decide if there paradigm could reflect an aversive conditioning.

I hope the authors can find time to assemble the knowledge around nicotine dosing for publication.

I learnt a lot from reading this paper and the ensuing efforts to address reviewers concerns.

**Response:**

We sincerely thank you for the encouraging feedback. We are glad that our revisions clarified the nicotine dosing and addressed your concerns. Your thoughtful comments and insights have been very helpful in improving the manuscript.
